# Juxtaglomerular Cell Tumor With Cytohistological Correlation: A Case Report

**DOI:** 10.1002/dc.25422

**Published:** 2024-11-20

**Authors:** Shane M. Woods, Robert Pei, Brant G. Wang

**Affiliations:** ^1^ University of Virginia School of Medicine Inova Campus Falls Church Virginia USA; ^2^ Department of Pathology Inova Fairfax Hospital Falls Church Virginia USA; ^3^ Department of Pathology Georgetown University Medical Center Washington DC USA; ^4^ Department of Pathology of Immunology Baylor College of Medicine Houston Texas USA

**Keywords:** cytology, immunohistochemistry, juxtaglomerular cell tumor

## Abstract

Juxtaglomerular cell tumors (JCT) are uncommon renin‐secreting tumors of the kidney with cytologic findings of JCT rarely reported. We describe a case of JCT in a 37‐year‐old man with uncontrolled hypertension that was cured by removal of the tumor via partial nephrectomy. Cytology material was prepared by scraping of the freshly sectioned tumor mass and stained with Diff‐Quik and Papanicolaou stains. Cytohistological findings and immunohistochemistry studies are discussed regarding diagnosis and differential diagnoses.

## 1 Introduction

Juxtaglomerular cell tumors (JCT), also known as reninomas, are a type of benign mesenchymal renin‐secreting tumor of the kidney, whereas metastasis is extremely rare [1, 2]. Though benign, clinically they usually manifest as uncontrolled hypertension with hypokalemia due to the effects of secondary hyperaldosteronism from dysregulation of the Renin‐Angiotensin‐Aldosterone axis [1, 2, 3, 4, 5, 6, 7]. These tumors are remarkably rare, first being described in 1967 by Robertson et al. [7]. In 1995 among a study population of 30,000 patients seen at a hypertension clinic, just 8 were observed to have reninomas [8]. Typically, patients affected are in their second and third decades of life with females more often affected than males [9].

Clinically, in addition to hypertension, associated symptoms are observed such as headaches, double vision or blurriness of vision, retinopathy, dizziness, polyuria, proteinuria, nausea, and vomiting [1, 2, 3, 4, 5, 6, 7, 8, 9, 10].

Common gross pathologic findings include a unilateral, well‐circumscribed, tan‐colored tumor with a fibrous capsule. On histology, sheets of ovoid or polygonal cells grow adjacent to renal tubules, with low presence of mitotic figures, but with rhomboid renin protogranules often present [1, 2, 4, 6, 9, 10, 11, 12, 13, 14].

Cytologic findings of JCT are extremely rare [14]. We describe scrape cytology of one such rare tumor.

### 1.1 Case Report

A 37‐year‐old man with a medical history of hypertension since childhood was not compliant with medications due to lack of insurance. He was found to have high renin and aldosterone, hypokalemia, and imaging findings of right lower pole renal mass, clinically suspected to be a reninoma. He had multiple emergency room visits and hospitalizations for hypertensive crisis with severe hypokalemia resulting in paralysis. The renal mass measured 3.2 cm compared to 2.3 cm 1 year prior. Due to worsening hypertensive crisis and increased size of the renal mass, partial nephrectomy was performed to remove the tumor. Presurgical renin and aldosterone levels measured up to 60 ng/mL/h and 30 ng/dL, respectively, whereas 1 day postsurgery, they dropped to 2.00 ng/mL/h and 3 ng/dL, respectively. His blood pressure also normalized after surgery.

**FIGURE 1:**
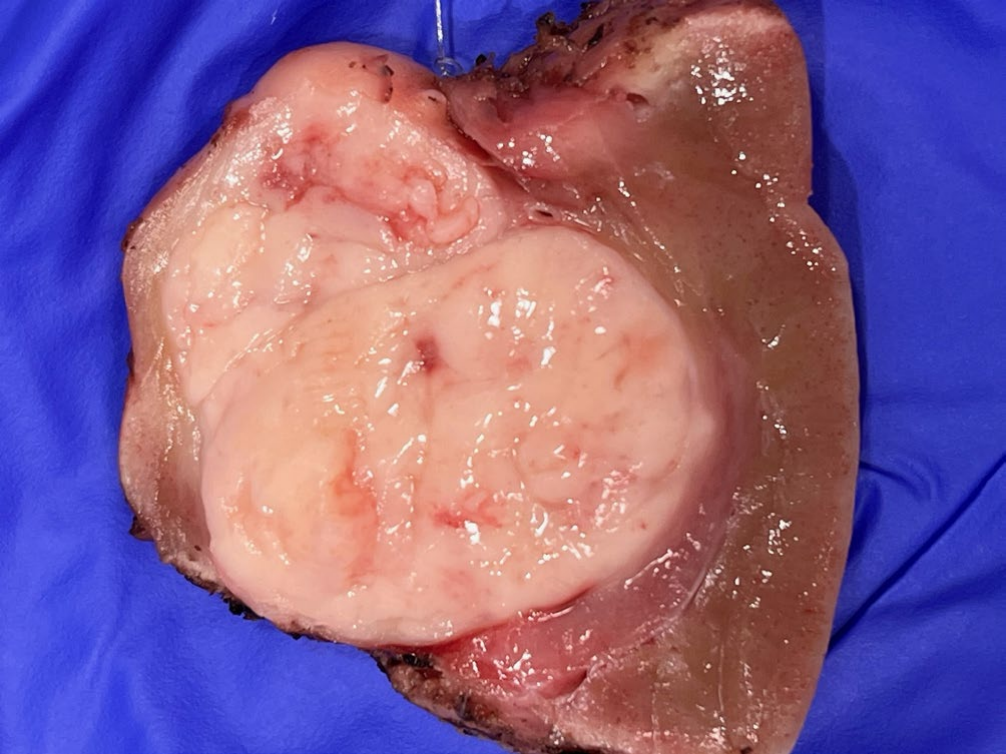
Gross examination of 3.2 cm renal tumor: Well‐circumscribed multi‐lobulated light yellow tan mass with no tumor necrosis or hemorrhage.

**FIGURE 2:**
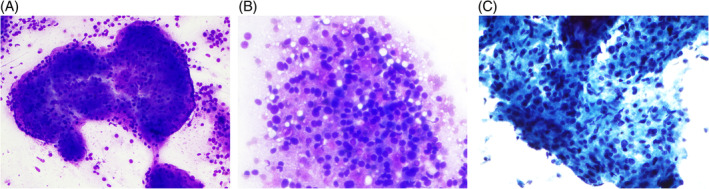
(A) Neoplastic cells in nested cohesive clusters or in single form. The spindle cells at edge of the nests (Diff‐Quik stain, ×200 magnification). (B) Neoplastic cells with nuclei showing mild polymorphism. Granular cytoplasm is easily stripped off. Rare spindled cells are present (Diff‐Quik stain, ×400 magnification). (C) Loosely cohesive cluster of neoplastic cells with nuclei showing even chromatin and indistinct nucleoli. Spindled cells can be appreciated (Papanicolaou stain, ×400 magnification).

Sectioning of the resected 3.2 cm right lower pole renal mass revealed light yellow tan, encapsulated, well‐circumscribed multilobated mass with no tumor necrosis or hemorrhage (Figure 1). Cytology material was obtained by scraping the fresh, sectioned mass and was deposited on glass slides and smeared over a separate slide. Diff‐Quik staining was performed on air‐dried smears. A portion of the tumor was chopped into small pieces and immersed in cytolyte preservative. Papanicolaou‐stained ThinPrep slides were prepared for microscopic examination. The majority of the mass was submitted into formalin for routine H&E examination. Diff‐Quik stained direct smears showed neoplastic cells in nested cohesive clusters or in single form (Figure 2A). Spindle cells were also seen at the edge of the nests. The neoplastic cells contained nuclei showing mild polymorphism and granular cytoplasm (Figure 2B). Papanicolaou‐stained ThinPrep slides showed loosely cohesive clusters of neoplastic cells with even chromatin and indistinct nucleoli. Spindled cells could be also appreciated (Figure 2C). Papillary structures were not identified.

**FIGURE 3:**
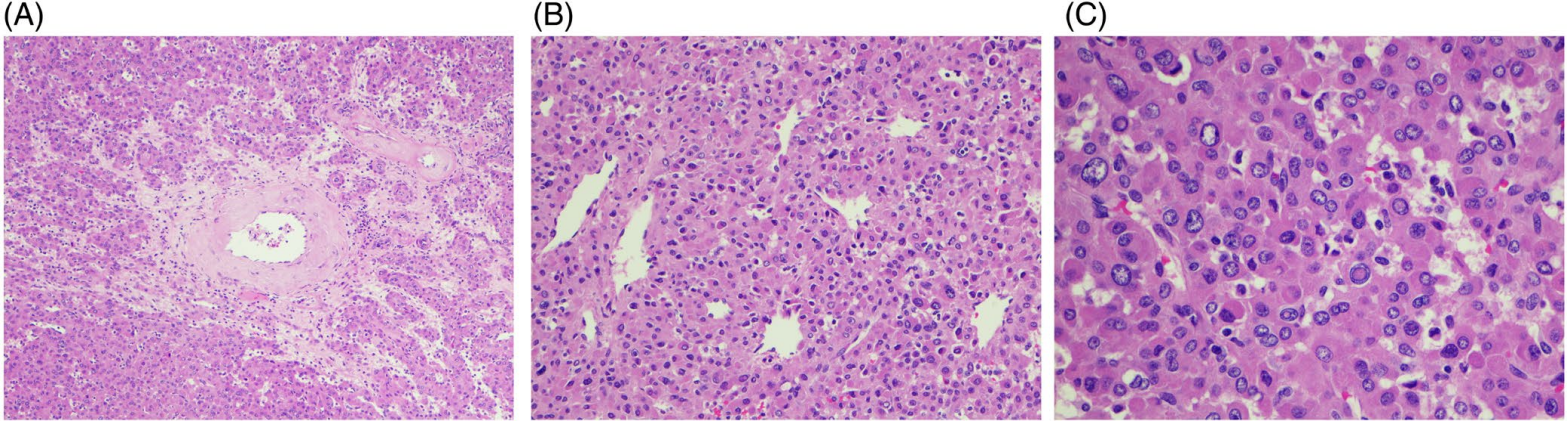
(A) Neoplastic cells in nests. Arterioles with concentric and hyalinized walls (hematoxylin and eosin stain, ×100 magnification). (B) Glomoid appearance with sheets of polygonal to oval cells with eosinophilic granular cytoplasm. Hemangiopericytoma‐like capillaries (hematoxylin and eosin stain, ×200 magnification). (C) Neoplastic cells with distinct cellular borders, mild nuclear polymorphism with occasional intranuclear pseudo‐inclusions, and abundant eosinophilic granular cytoplasm. Mitotic figures and tumor necrosis are not identified (hematoxylin and eosin stain, ×400 magnification).

**FIGURE 4:**
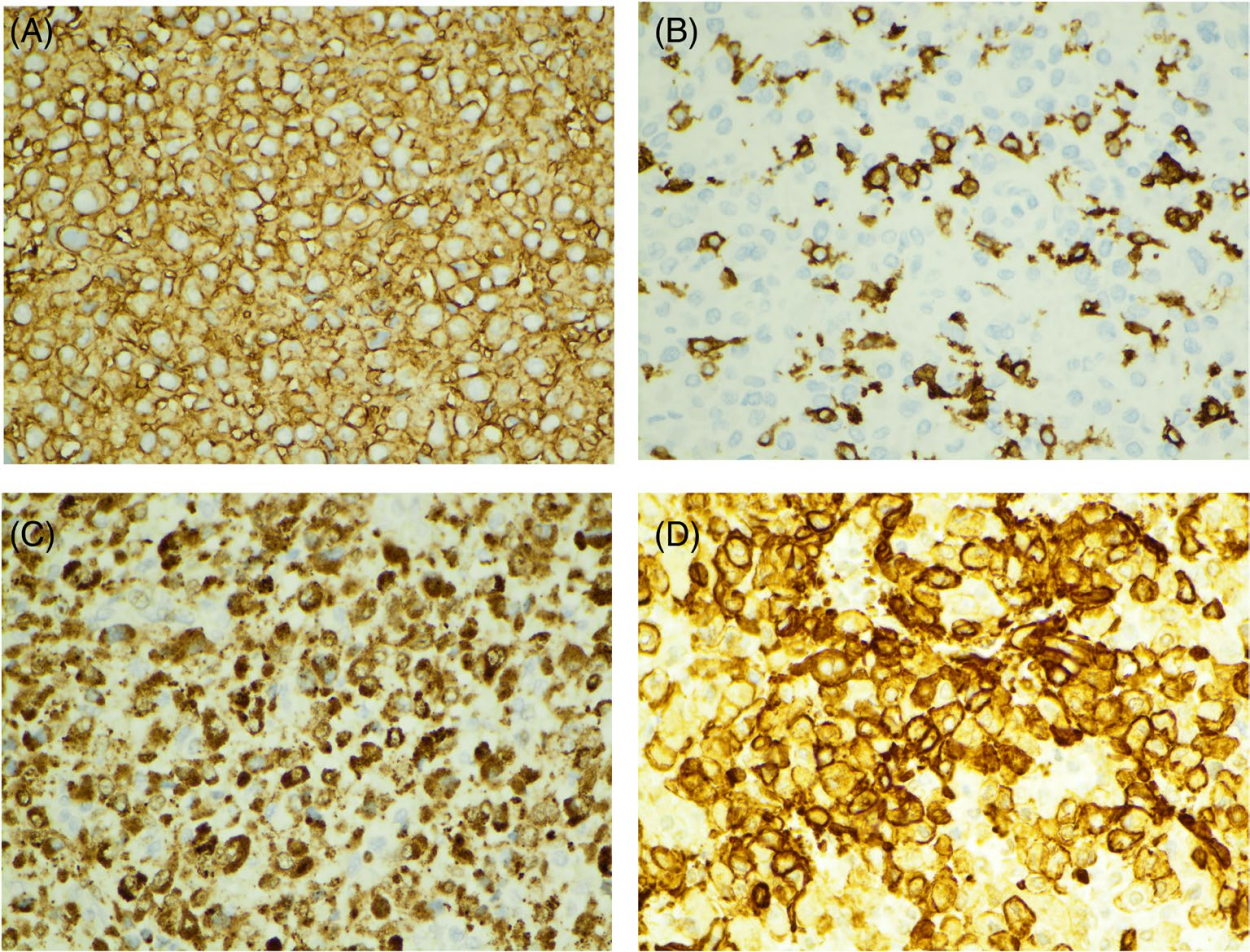
(A) Neoplastic cells showing cytoplasmic positivity for CD34 immunostain (×400 magnification). (B) Neoplastic cells showing focal cytoplasmic positivity for CD117 immunostain (×400 magnification). (C) Neoplastic cells showing nuclear and cytoplasmic positivity for GATA3 immunostain (×400 magnification). (D) Neoplastic cells showing cytoplasmic positivity for smooth muscle actin (×400 magnification).

H&E‐stained slides showed polygonal neoplastic cells arranged in nests. Arterioles with concentric and hyalinized walls suggestive of hypertensive effect were readily identified (Figure 3A). Prominent hemangiopericytoma‐like capillaries were present (Figure 3B). The neoplastic cells demonstrated mild nuclear pleomorphism and granular eosinophilic cytoplasm. No tumor necrosis, hemorrhage, or mitotic figures were identified. Occasional intranuclear pseudo‐inclusions were seen (Figure 3C). Immunostains showed that neoplastic cells were positive for CD34 (Figure 4A), CD117 (focal, Figure 4B), GATA3 (Figure 4C), smooth muscle actin (Figure 4D), and vimentin, and were negative for pancytokeratin, PAX8, DOG1, INSM1, synaptophysin, HMB45, and Melan‐A. The findings supported the diagnosis of juxtaglomerular cell tumor.

## 2 Discussion

The relative rarity of reninomas have produced only limited cytology reports [14]. This scarcity of literature coupled with the heterogeneity of cytologic features can make a cytologic diagnosis challenging if clinical correlation is absent. Histological patterns observed for JCTs include, but are not limited to, sheets, trabeculae, microcysts, tubular structures, and papillae [1, 2, 4, 6, 9, 10, 11, 12, 13, 14]. Atypical pathological features, such as marked pleomorphism and rhabdoid morphology, were not present with this case.

The differential diagnosis for a JCT includes common renal tumors, like renal cell carcinomas, oncocytomas, or angiomyolipomas, and also includes less common entities like renal glomus tumors, pheochromocytomas, or even primary renal endocrine tumors [1, 2, 9, 10, 11, 12, 13, 14, 15, 16, 17, 18]. Cytology and histology, though not specific, can narrow down the differential diagnoses, and immunostains can be helpful for definitive diagnosis. In this case, negative immunoreactivity for pancytokeratin and PAX8 as well as lack of more dedicated vasculature excluded the diagnosis of renal cell carcinoma. Negative immunoreactivity for pancytokeratin and PAX8 also excluded the diagnosis of renal oncocytoma. Negative immunoreactivity for HMB45 and Melan‐A excluded the diagnosis of angiomyolipoma. GATA3 immunoreactivity can be seen in paragangliomas or pheochromocytomas. However, endocrine differentiation was not documented by immunostains. The glomoid appearance and hemangiopericytoma‐like capillaries, especially smooth muscle actin positivity can be confused with a pericytic tumor of the kidney such as renal glomus tumor [16, 17]. However, glomus tumors are negative for CD34, GATA3, and CD117. Positive immunoreactivity for CD34 and CD117 raised the possibility of a gastrointestinal stromal tumor (GIST). However, the neoplastic cells were negative for DOG1. In addition, the clinical presentation of a renal mass does not align with that of a GIST. Renin is one specific immunostain for confirming the diagnosis of JCT but we were unable to identify a commercial laboratory that provides this immunohistochemistry service. Ultimately the differential diagnoses can be excluded when incorporating the clinical presentation of hypertension, elevated renin, aldosterone, and hypokalemia, in addition to the findings of normalized renin and aldosterone levels, and normalized blood pressure levels after removal of the tumor.

One major limitation of this report is that the cytology material prepared for this case is from a scraping of the freshly resected tumor, rather than a fine needle aspiration (FNA). A JCT is a rare tumor and FNA is seldom used to diagnose a JCT prior to resection [14]. The heterogeneity of cytologic features of JCT can make a cytologic identification challenging, and characteristic clinical findings more often guide diagnosis. Potential contraindications of FNA of a JCT include tumor seeding in cases with malignant tumor, bleeding in cases with a vascular‐rich tumor, and elevated blood pressure in cases associated with a paraganglioma or pheochromocytoma. We were interested in describing specific cytomorphological features of a JCT when we were made aware of a potential resection of a JCT. Following the resection, we scraped the tumor and obtained material for cytology. Compared to FNA, scraping is done after resection and it obtains more cellular material, covers a larger surface area but with fewer discohesive cells. Scraping can aid intraoperative pathology consultation during surgery. We did not have FNA material of the tumor described in this case, thus we were not able to compare FNA cytology and scraping cytology of the same tumor to delineate the subtle difference between FNA and scraping cytology. Compared to a previous report [14], this case further confirms the heterogeneity of cytologic features of JCTs.

## Conflicts of Interest

The authors declare no conflicts of interest.

## Data Availability

The data that support the findings of this study are available on request from the corresponding author. The data are not publicly available due to privacy or ethical restrictions.
